# Insights on the Health Benefits and Functional Potential of Food Bioactive Compounds

**DOI:** 10.3390/foods14111984

**Published:** 2025-06-04

**Authors:** Diego Morales

**Affiliations:** Departmental Section of Galenic Pharmacy and Food Technology, Veterinary Faculty, Complutense University of Madrid, 28040 Madrid, Spain; diego.morales@ucm.es

In recent decades, the global scientific community has shown a growing interest in the field of functional foods and bioactive compounds, spurred by a confluence of public health challenges and emerging nutritional strategies. The rising prevalence of chronic non-communicable diseases such as cardiovascular disease, cancer, and neurodegenerative disorders has positioned diet as a central pillar of both prevention and intervention [1,2]. There is also increasing societal awareness of the link between diet and health, driven by a surge in scientific evidence demonstrating how dietary patterns and specific food molecules influence the onset and progression of chronic diseases. As academic dissemination, health campaigns, and media coverage continue to increase public knowledge, individuals are recognizing more and more the role of nutrition in reducing the risk of certain disorders and promoting overall well-being and quality of life [3,4,5], thereby boosting consumer demand for functional foods and nutraceuticals [6,7].

Targeting these desirable bioactive compounds, which may help reduce the aforementioned risks and alleviate certain signs and symptoms, researchers have explored a plethora of natural sources and food matrices to develop novel functional foods. In this context, green and advanced extraction technologies are commonly employed to obtain enriched fractions or isolated/purified bioactive ingredients. In this vein, the use of supercritical fluids, pressurized liquids, deep eutectic solvents, and ionic liquids, along with extraction protocols assisted by microwaves, ultrasound, and pulsed electric fields, is increasingly prevalent in both food research and the food industry [8,9]. Additionally, methodologies such as microbial fermentation and enzymatic hydrolysis are often used to enable molecular transformation, synthesis, and/or the release of bioactives [10,11].

Furthermore, the extracts and fractions obtained need to be thoroughly characterized, and their biological activities must be evaluated both in vitro and in vivo—not only using animal models but also through clinical trials. The most widely studied bioactivities include antioxidant, anti-inflammatory, anti-proliferative, hypolipidemic, hypocholesterolemic, hypoglycemic, antihypertensive, antimicrobial, and prebiotic properties [12,13]. Two limitations in functional food research worth emphasizing are the limited number of published clinical trials and the lack of correlation studies linking specific molecules to health outcomes. Surprisingly, there is also a dearth of sensory analyses and consumer acceptance studies, which are essential to ensure that these potentially health-promoting products can be successfully integrated into the food market [14,15]. Therefore, further investigation along these underexplored lines should be strongly encouraged.

Consequently, the main aim of this Special Issue (SI) is to present the latest findings and insights related to the health benefits and functional potential of food-based bioactive compounds. This SI comprises two review articles and nine original research papers that collectively address current knowledge, existing limitations, recent advances, and future perspectives within this field.

One of the review articles in this SI surveyed the literature on the promising anticancer bioactive ingredients found in food, such as thymoquinone, allicin, resveratrol, parthenolide, epigallocatechin gallate, and piperine, targeting specific cancer hallmarks, such as sustained proliferation, apoptosis inactivation, the stimulation of angiogenesis, immune evasion, and altered metabolism [16]. The other reviewed the published articles evaluating the potential health effects of strawberry tree (*Arbutus unedo*) fractions and extracts, considering their biological activities, the parts of the plant used (fruit, leaves, flowers, honey, roots, wood, etc.), the experimental models, the target compounds, and the extraction methods applied [17].

The original research articles investigated a wide range of natural and food sources, extraction methodologies, biological activities, and experimental models. For instance, Han et al. (2025) observed that naringenin—a food bioactive compound present in tomatoes and citrus fruits—exerted in vitro anti-inflammatory effects, as it was able to reduce oncostatin M release and mRNA expression in neutrophil-like differentiated HL-60 cells [18]. Jimenez-Pulido et al. (2024) also reported related activities, in this case synergistic anti-inflammatory and antioxidant properties in cereal (wheat and oat)-based nutraceutical formulas combining extracts and hydrolysates from sprouts and byproducts. Certain resulting mixtures exhibited in vitro radical-scavenging activity and inhibited pro-inflammatory cytokine secretion in murine macrophages. The total phenolic, β-glucan, and avenanthramide contents in the blends appeared to be crucial for their biological activities [19]. Cantero-Bahillo et al. (2024) applied hydrolysis strategies to obtain bioactive co-extracts from fenugreek seeds and quinoa husk, identified as steroidal- and terpenoid-rich saponins sources, respectively. Pancreatic lipase inhibition, interference with cholesterol bio-accessibility, and reductions in cytokine release were revealed through in vitro approaches [20]. Hypoglycemic and antidiabetic effects were likewise reported in two other contributions included in this SI: Tejedor-Calvo et al. (2023) applied pressurized liquid technology using different proportions of water and ethanol to obtain extracts from several truffle species. These fractions were rich in β-glucans, chitins, heteropolysaccharides, fatty acids, phenolic compounds, and other bioactives. Specific truffle extracts inhibited enzymes involved in type 2 diabetes; α-amylase and α-glucosidase activities were reduced by aqueous and ethanolic fractions, respectively [21]. Similar inhibitory effects on these enzymes were noticed in fractions collected by applying ultrasound-assisted extractions to the skin of different grapes of indigenous Croatian white grapevine varieties. The obtained extracts also showed antioxidant capacity against DPPH^●^ [22]. Another relevant in vitro study was conducted by Gallegos-Alcalá et al. (2023), who found that glycomacropeptide, a milk-derived bioactive peptide, provided protection against inflammation and oxidative stress in an in vitro model of atopic dermatitis using human keratinocytes. It also promoted wound healing [23].

In addition to these in vitro findings, several studies validating biological activities in animal models are included in this SI. Liu et al. (2024) demonstrated that supplementation with procyanidin B1 and coumaric acid from highland barley alleviated high-fat-diet-induced hyperlipidemia in diabetic C57BL/6J mice. This supplementation regulated PPARα-mediated hepatic lipid metabolism, resulting in reductions in total cholesterol, triglyceride, and LDL-cholesterol levels and increased HDL-cholesterol levels. Furthermore, an amelioration of gut microbiota dysbiosis was observed [24]. C57BL/6J mice were also used by Chu et al. (2023): glycated casein—produced via transglutaminase-type—exerted protective effects against dextran sulfate sodium (DSS)-induced intestinal inflammation. This intervention led to reductions in weight, disease activity index scores, colon length, colonic mucosal damage, and levels of pro-inflammatory markers in both serum and colons. Moreover, glycated casein modulated the expression of proteins involved in the TLR4/NF-κB signaling pathway. The authors concluded that it showed a greater protective effect against DSS-induced colitis than native casein [25]. Additionally, Min et al. (2023) investigated the effects of *Aquilaria sinensis* (a medicinal plant widely grown in South China) leaf supplementation on lipid metabolism in castrated male Chinese goats. The results revealed significant decreases in blood cholesterol levels along with increased HDL concentrations. Metabolomics analysis confirmed cholesterol levels had reduced in both serum and muscle tissue [26].

Before concluding this editorial, I would like to express my sincere gratitude to all the authors and reviewers who contributed to this SI as well as *Foods* and the entire MDPI team for their support and for granting me the opportunity to serve as Guest Editor. I hope this collection of studies helps increase knowledge in the field of functional foods and bioactive compounds and that it inspires further research in order to address existing gaps and advance scientific understanding in this relevant area.
